# Targeted Toxin-Based Selectable Drug-Free Enrichment of Mammalian Cells with High Transgene Expression

**DOI:** 10.3390/biology2010341

**Published:** 2013-02-28

**Authors:** Masahiro Sato, Eri Akasaka, Issei Saitoh, Masato Ohtsuka, Shingo Nakamura, Takayuki Sakurai, Satoshi Watanabe

**Affiliations:** 1 Section of Gene Expression Regulation, Frontier Science Research Center, Kagoshima University, Kagoshima 890-8544, Japan; 2 Department of Pediatric Dentistry, Graduate School of Medical and Dental Sciences, Kagoshima University, Kagoshima 890-8544, Japan; E-Mails: stylistics777@yahoo.co.jp (E.A.); isaito@dent.niigata-u.ac.jp (I.S.); 3 Division of Basic Molecular Science and Molecular Medicine, School of Medicine, Tokai University, Kanagawa 259-1193, Japan; E-Mail: masato@is.icc.u-tokai.ac.jp; 4 Department of Surgery, National Defense Medical College, Saitama 359-8513, Japan; E-Mail: snaka@ndmc.ac.jp; 5 Department of Organ Regeneration, Graduate School of Medicine, Shinshu University, Nagano 390-8621, Japan; E-Mail: tsakurai@shinshu-u.ac.jp; 6 Animal Genome Research Unit, Division of Animal Science, National Institute of Agrobiological Sciences, Ibaraki 305-8602, Japan; E-Mail: kettle@affrc.go.jp

**Keywords:** α-Gal epitope, BS-I-B_4_ lectin, endo-β-galactosidase C, porcine embryonic fibroblasts, saporin, targeted toxin, high transgene expression

## Abstract

Almost all transfection protocols for mammalian cells use a drug resistance gene for the selection of transfected cells. However, it always requires the characterization of each isolated clone regarding transgene expression, which is time-consuming and labor-intensive. In the current study, we developed a novel method to selectively isolate clones with high transgene expression without drug selection. Porcine embryonic fibroblasts were transfected with pCEIEnd, an expression vector that simultaneously expresses enhanced green fluorescent protein (EGFP) and endo-β-galactosidase C(EndoGalC; an enzyme capable of digesting cell surface α-Gal epitope) upon transfection. After transfection, the surviving cells were briefly treated with IB4SAP (α-Gal epitope-specific BS-I-B_4_ lectin conjugated with a toxin saporin). The treated cells were then allowed to grow in normal medium, during which only cells strongly expressing EndoGalC and EGFP would survive because of the absence of α-Gal epitopes on their cell surface. Almost all the surviving colonies after IB4SAP treatment were in fact negative for BS-I-B_4_ staining, and also strongly expressed EGFP. This system would be particularly valuable for researchers who wish to perform large-scale production of therapeutically important recombinant proteins.

## 1. Introduction

Transfection of mammalian cells with exogenous DNA (termed “transgene”) has long been used as a powerful experimental tool to evaluate the properties and functions of newly isolated genes. This technology has also been proven useful for generating “cell factories” for large-scale production of recombinant proteins used in the fields of pharmacology and medicine. Vectors that are generally used as a vehicle for the delivery of a transgene into cells are largely divided into two types, namely, viral and non-viral (plasmid) vectors; the latter has been widely used by researchers because of convenience in plasmid preparation and transfection.

In most plasmid-based transfection experiments, the vectors contain a selectable marker gene (such as neomycin resistance gene [*neo*]) that confers resistance against a specific drug. After transfection of these vectors, the cells have to be maintained in the presence of the drug to enrich the transfectants for approximately one week. The transfectants carrying a selectable marker gene express protein products that destroy (detoxify) the drug, whereas non-transfectants do not. However, the expression levels of a selectable marker gene and, probably, a gene of interest (GOI) among the transfectants are variable. Therefore, to obtain transfectants with high transgene expression, the levels of the introduced transgene in individual isolated colonies have to be investigated independently or the cells need to be segregated, for example, by fluorescence-activated cell sorting. These steps are not only laborious but also time-consuming. Moreover, this selectable marker-based system cannot be used for cells exhibiting multidrug resistance, and therefore, a new drug-free system for the selection of cells with high transgene expression has long been awaited.

Almost all mammalian cells, except those from humans and Old World apes, express Galα1-3Gal (an α-Gal epitope) on their cell surface [1,2,3,4]. The α-Gal epitope is synthesized via cell surface-localized α-1,3-galactosyltransferase (α-GalT) and is a causative agent for hyperacute rejection upon pig-to-human xenotransplantation [5]. The *Clostridium perfringens*-derived endo-β-galactosidase C (EndoGalC) is known to digest the α-Gal epitope [6,7]. Therefore, introduction of an EndoGalC construct into the porcine genome has been considered as a promising approach to generate genetically modified piglets suitable for xenotransplantation [7,8,9]. In addition, the absence of an α-Gal epitope can be easily monitored by staining cells with *Bandeiraea simplicifolia* isolectin-B_4_ (BS-I-B_4_, IB4), a lectin that specifically binds to the α-Gal epitope [1].

**Figure 1:**
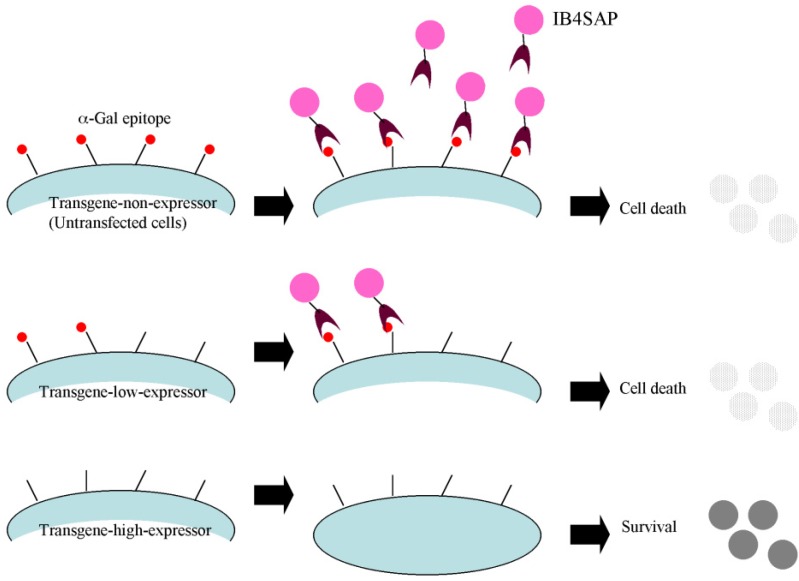
Schematic diagram of a mechanism for targeted toxin-mediated drug-free isolation of cells with high transgene expression. The untransfected cells (“transgene non-expressors”) expressing the α-Gal epitope on their surface are targeted by IB4SAP, which subsequently leads to cell death. When the cells are transfected with a vector expressing EndoGalC that digests the α-Gal epitope, the cells weakly expressing EndoGalC (“transgene low-expressors”) will still be killed by IB4SAP through binding to the residual α-Gal epitope on the cell surface. In contrast, the cells strongly expressing EndoGalC (“transgene high-expressors”) will survive IB4SAP treatment because of the complete loss of the α-Gal epitope on their surfaces.

Targeted toxins consist of the ribosome-inactivating protein saporin (SAP) [10] that is conjugated to a target molecule recognizing a cell-specific marker. When administered to the cells of interest, the conjugate binds to, and is absorbed by, the target cells, which results in the release of SAP and subsequent ribosome inactivation. In contrast, the cells not expressing the target molecule do not bind or absorb the conjugate and are not affected. Therefore, targeted toxins have been considered as a powerful tool for removing unwanted cells from a pool of genetically modified population. In fact, we have previously demonstrated successful application of this technology for the isolation of transfectants with high transgene expression from among porcine embryonic fibroblasts (PEFs) transfected with the EndoGalC construct [8]. Moreover, the elimination of unwanted cells, including those that are untransfected and those weakly expressing the α-Gal epitope (considered as cells with low transgene expression), can be performed simply by incubating the target cells with SAP-conjugated IB4 (hereafter referred to as “IB4SAP”) for a short period, followed by culture under normal conditions. As expected, the surviving cells are those that do not express the α-Gal epitope on their cell surface. Based on these findings, we propose that coexpression of a gene of interest and EndoGalC, along with subsequent IB4SAP treatment, as depicted in Figure 1, would result in enrichment of α-Gal epitope-negative cells that strongly express GOI.

In the current study, we examined whether the EndoGalC/IB4SAP-based selection system is effective for the isolation of “transgene high-expressors”.

## 2. Results and Discussion

### 2.1. Experiment 1: Inverse Relationship between EndoGalC and α-Gal Epitope Expression

**Figure 2:**
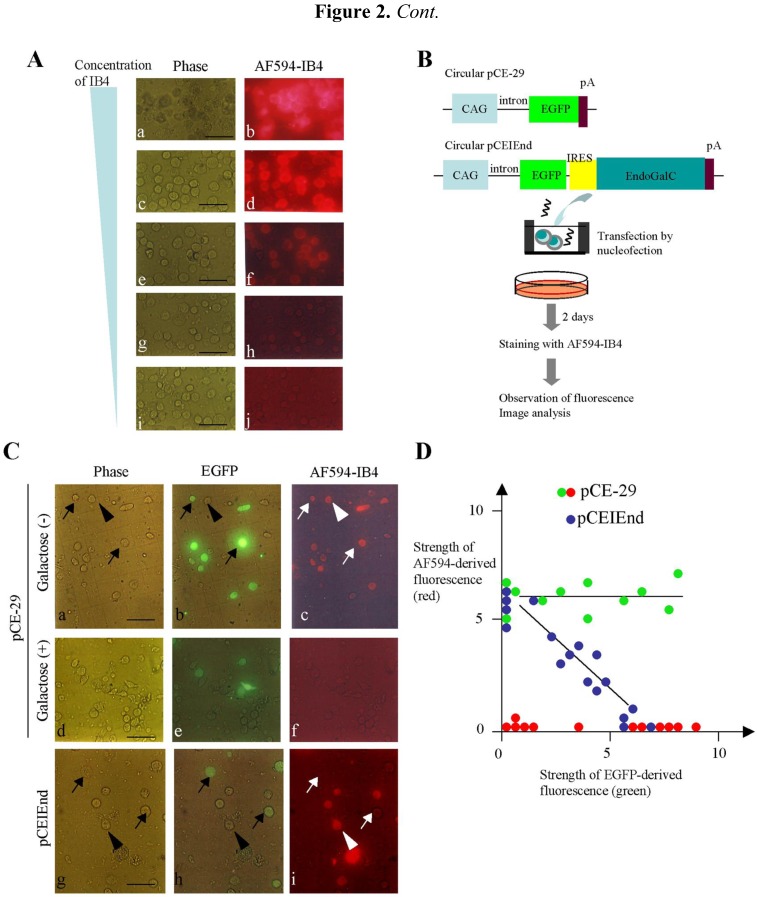
(**A**) Staining of PEFs with various concentrations [50 (**a**, **b**), 10 (**c**, **d**), 2 (**e**, **f**), 0.4 (**g**, **h**) and 0.08 (**i**, **j**) μg/mL] of AF594-IB4. Note strong reactivity in the cells stained with 50 to 10 μg/mL of AF594-IB4 (**a**–**d**). (**B**) Expression constructs and experimental flow for examination of the relationship between EndoGalC and α-Gal epitope expression. CAG, approximately 1-kb cytomegalovirus enhancer with chicken β-actin promoter and its 1st intron; EGFP, 0.9-kb enhanced green fluorescent protein; IRES, 0.63-kb internal ribosomal entry site; EndoGalC, 3-kb *C. perfringens*-derived endo-β-galactosidase C; and pA, 0.56-kb poly(A) sites of rabbit β-globin gene. (**C**) Cytochemical staining of transfected PEFs with AF594-IB4. Note that the PEFs transfected with pCE-29 were uniformly stained with the lectin, irrespective of the strength of EGFP fluorescence (indicated by arrows and the arrowhead in **a**–**c**). In contrast, in the case of transfection with pCEIEnd, PEFs not expressing or weakly expressing EGFP were distinctly stained by the lectin (indicated by the arrowhead in **g**–**i**), while PEFs relatively strongly expressing EGFP were almost negative for the staining (indicated by arrows in **g**–**i**), suggesting complete loss of the α-Gal epitope from their surface. Staining of the pCE-29-introduced PEFs with AF594-IB4 + galactose abolished the staining completely (**d**–**f**). Phase (**a**, **d**, **g**), photographs taken under light; EGFP **(b**, **e**, **h**), photographs taken under light + UV illumination to detect EGFP-derived green fluorescence; and AF594-IB4 (**c**, **f**, **i**), photographs taken under light + UV illumination to detect AF594-derived red fluorescence. Bar = 50 μm. (**D**) Image analysis of the transfected PEFs shown in (**C**). The intensity of each cell was measured and plotted, with the arbitrary fluorescence intensity shown in both the abscissa and ordinate axes. The green and blue dots indicate fluorescence measured from the AF594-IB4-stained cells that were transfected with pCE-29 and pCEIEnd, respectively. The red dots indicate fluorescence from the pCE-29-transfected cells that were stained with AF594-IB4 + 50 mM galactose.

As a preliminary test, PEFs were stained with the serially diluted Alexa Fluor 594-labeled IB4 (hereafter referred to as “AF594-IB4”) to know the optimal concentration of AF594-IB4 exhibiting strong binding to the cells. As shown in Figure 2A, 50–10 μg/mL of AF594-IB4 were found to be highly reactive to the PEFs. Two μg/mL of AF594-IB4 yielded moderate staining for α-Gal epitope expression. Therefore, we hereafter decided to use more than 50 μg/mL of IB4SAP for isolation of α-Gal epitope-negative transfectants.

To explore the relationship between the EndoGalC and α-Gal epitope expression, we transfected PEFs with the pCEIEnd plasmid (Figure 2B), which expresses enhanced green fluorescent protein (EGFP) and EndoGalC simultaneously because of the presence of internal ribosomal entry site (IRES) [11,12,13] between the EGFP and EndoGalC genes. PEFs transfected with pCE-29 plasmid were used as the control (Figure 2B). At two days after transfection, the cells collected by trypsinization were stained with AF594-IB4. In the case of pCEIEnd transfection, the cells strongly expressing EGFP were almost completely negative for IB4 staining (Figure 2C, arrows in g–i), whereas those not expressing or weakly expressing EGFP showed distinct staining (Figure 2C, arrowheads in g–i). In the case of pCE-29 transfection, all the cells were stained with lectin, irrespective of EGFP expression (Figure 2C, arrows and arrowheads in a–c). However, incubation of the pCE-29-transfected cells with AF594-IB4 + 50 mM galactose abolished the IB4-specific staining (d–f in Figure 2C). The image analysis confirmed these observations (green dots *vs*. red dots in Figure 2D). Also, there was an inverse relationship between EndoGalC and α-Gal epitope expression in the pCEIEnd-transfected cells (blue dots in Figure 2D). Thus, transgene high-expressors exhibited greatly reduced expression levels of the α-Gal epitope.

### 2.2. Experiment 2: Enrichment of Transgene High-Expressors with Toxin-Conjugated IB4 Treatment

**Figure 3:**
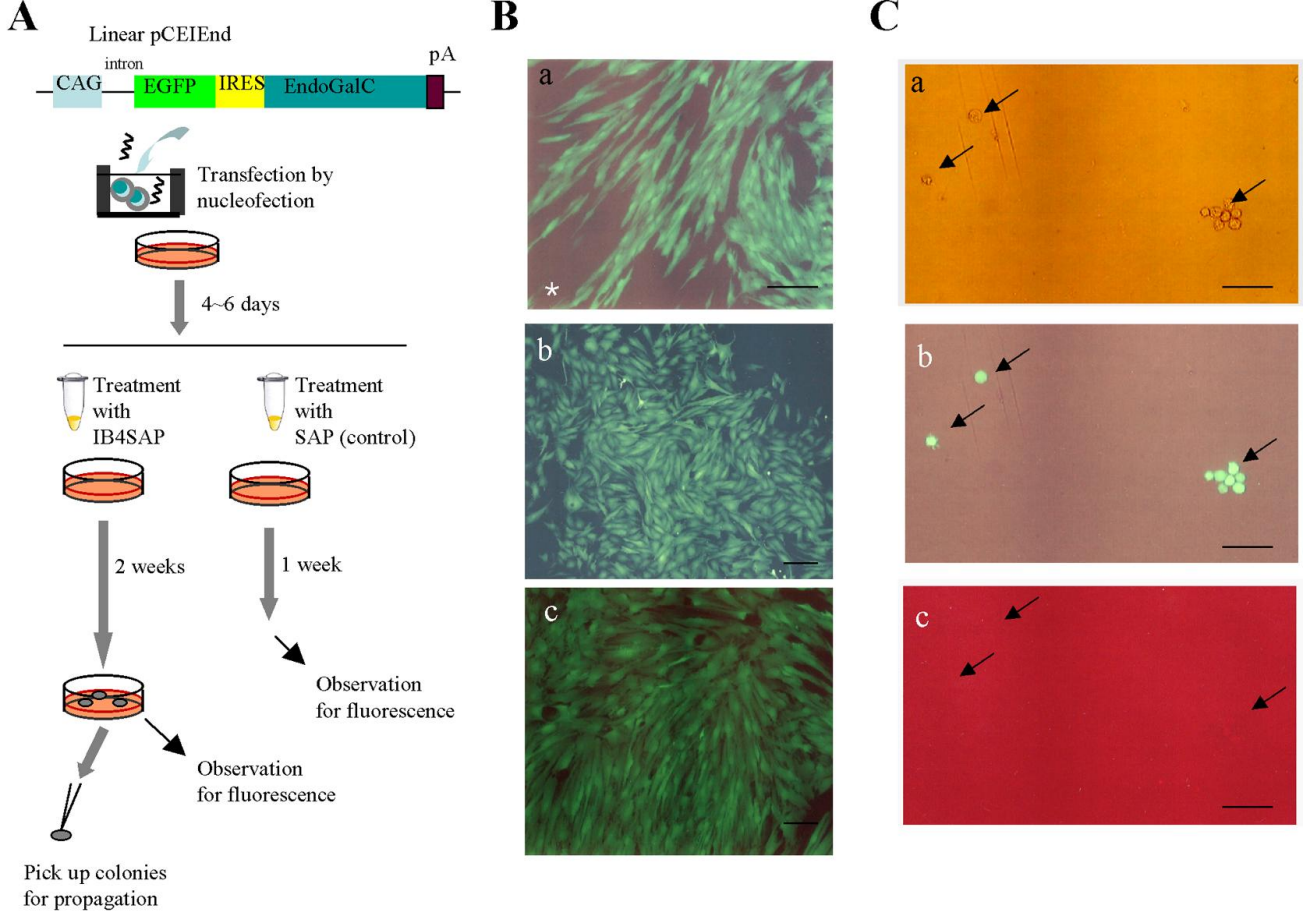
(**A**) Experimental flow for examining whether enrichment of cells with high transgene expression can be achieved with IB4SAP treatment. Abbreviations are the same in Figure 2A. (**B**) Micrographs taken under light + UV illumination. (**a**), The cells obtained one week after SAP treatment; and (**b**) and (**c**), the emerging colonies obtained two weeks after IB4SAP treatment. In (**a**), a mixture of fluorescent cells (successfully transfected with pCEIEnd) and nonfluorescent untransfected cells is indicated by an asterisk. The cells in (**b**) and (**c**) are from two independent colonies. Bar = 100 μm. (**C**) Dissociated cells isolated from a colony described in (**c**) of **B**. These cells were then stained with AF594-IB4. The arrows indicate the isolated cells in (**a**) showing bright green fluorescence (**b**) but negative for lectin staining (**c**). (**a**), photograph taken under light; (**b**) and (**c**), photographs taken under light + UV illumination. Bar = 100 μm.

Next, we performed IB4SAP treatment to enrich transgene high-expressors exhibiting distinct EGFP fluorescence, but reduced expression levels of the α-Gal epitope. As depicted in Figure 3A, PEFs transfected with pCEIEnd were cultured for four to six days in normal medium (without drug selection). The cells were subsequently split into two groups: one treated with IB4SAP and the other with SAP (control). After treatment, the cells were reseeded in a 60-mm tissue culture dish containing normal medium. Within two days, extensive cell death was observed in the IB4SAP-treated group, in contrast to the SAP-treated cells (data not shown). One week after reseeding, the cells in the control group reached confluency and were found to contain a mixture of fluorescent and non-fluorescent cells determined by a fluorescence microscope [Figure 3B(a)]. In contrast, almost all the emerging colonies obtained two weeks after IB4SAP treatment exhibited bright and strong EGFP-derived green fluorescence [Figure 3B(b,c)], even though there were some nonfluorescent colonies (Table 1). Subsequently, some of these fluorescent colonies were subjected to staining with AF594-IB4. All cells exhibited green fluorescence [Figure 3C(b), arrows] but lost AF594-derived red fluorescence [Figure 3C(c), arrows], indicating greatly reduced levels of the α-Gal epitope on their surface. Long-term (more than six months; over 40 passages) cultivation did not alter their phenotype (high levels of EGFP expression and greatly reduced levels of the α-Gal epitope expression) (data not shown). Moreover, staining of nonfluorescent colonies with lectin resulted in the appearance of red fluorescence (data not shown), suggesting that they were likely to be non-transfectants that survived after IB4SAP treatment. We also found that these nonfluorescent cells could be excluded by repeated treatment with IB4SAP (data not shown).

### 2.3. Experiment 3: Targeted Toxin-Based Enrichment of Transgene High-Expressors Is Applicable to Multidrug-Resistant Cells

**Figure 4:**
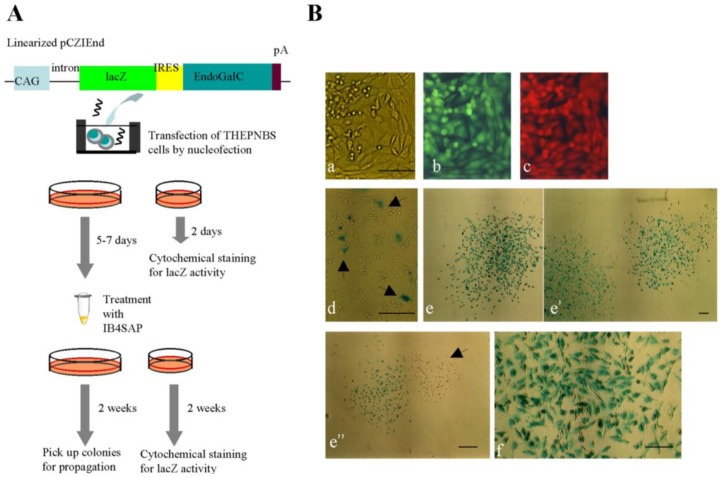
(**A**) Experimental flow for examining whether enrichment of cells with high transgene expression in the multidrug-resistant cells (THEPNBS). (**B**) The THEPNBS cells (**a**–**c**) express both EGFP-derived green fluorescence (**b**) and tdTomato-derived red fluorescence (**c**) under light + UV illumination. (**d**) Cytochemical staining of THEPNBS cells for lacZ activity at two day after transfection. The arrowheads indicate the cells exhibiting blue deposits in their cytoplasm, while the other cells were negative for such staining, probably reflecting unsuccessful transfection in those cells. (**e**–**e''**) Two weeks after IB4SAP treatment, the colonies were stained for lacZ activity. An arrowhead in (**e''**) indicates some colonies with no staining for lacZ activity (**f**) Magnified view of a stained colony demonstrates uniform distribution of the lacZ protein throughout the cells. Bar = 100 μm.

To extend the usefulness of this novel system for multidrug-resistant cells, we introduced a transgene (Figure 4A) into a multidrug-resistant porcine cell line THEPNBS, which carries two fluorescent marker genes (EGFP and tdTomato) as well as five drug resistance genes (*puro*, *neo*, *hyg*, *Sh ble*, and *zeo*) [14]. As expected, simultaneous expression of EGFP and tdTomato was observed in the parental THEPNBS cells [Figure 4B(b,c)]. As depicted in Figure 4A, the THEPNBS cells were transfected with pCZIEnd carrying the lacZ gene that codes for *E. coli*-derived β-galactosidase, which can be easily detected by cytochemical staining with its substrate, X-Gal. At two days after transfection, only a few cells exhibited lacZ activity [Figure 4B(d), arrowheads], whereas the majority of cells had negative results for staining [Figure 4B(d)]. At five to seven days after transfection, the cells harvested by trypsinization were subjected to IB4SAP treatment and then cultured in normal medium. Two weeks after reseeding, the emerging colonies were fixed and examined by cytochemical staining for lacZ activity. As expected, almost all the colonies (26/30) obtained were distinctly stained [Figure 4B(e,e’)], and higher magnification also showed uniform distribution of lacZ activity throughout the colonies [Figure 4B(f)]. However, a few colonies (4/30) were negative for X-Gal staining [Figure 4B(e’’), arrowhead]. As mentioned previously, these colonies may be derived from non-transfectants that survived after IB4SAP treatment. Nevertheless, our results demonstrate that this IB4SAP-based drug-free selection system is applicable to multidrug-resistant cells.

**Table 1:** Summary of Experiment 2.

Experiment ^1^	No. of colonies generated after IB4SAP treatment	No. of colonies inspected for EGFP fluorescence	No. colonies with various degrees of fluorescence ^2^			
			++	+	+/−	−
1	20	15	11	2	0	2
2	33	24	20	3	0	1
3	14	10	10	0	0	0

^1^ As depicted in Figure 3A, the number of colonies emerging after transfection with pCEIEnd DNA and subsequent treatment with IB4SAP was recorded. Some colonies were inspected for EGFP-derived green fluorescence under a stereomicroscope and scored based on the strength of fluorescence. Experiment was performed in each different day. ^2^ The strength of fluorescence was classified as ++ (strong), + (moderate), +/− (faint) and − (no fluorescence).

Targeted toxin technology using IB4SAP was first applied for specific elimination of porcine cells that were untransfected, or weakly expressed the EndoGalC gene, to create genetically modified cells suitable for pig-to-human xenotransplantation [8]. On the basis of this previous study, we decided to utilize this novel system for selecting transgene high-expressors, as described in Figure 1. Here, we confirm that this system works well. Unfortunately, this system is only applicable to mammalian cells that express the α-Gal epitope. Human and Old World monkey cells that do not express such an epitope due to mutations in the α-GalT gene [15] cannot be used. Theoretically, if these α-Gal epitope-negative cells are genetically engineered to express α-GalT, then the system would become applicable.

The present system is based on the coexpression of the EndoGalC gene and a GOI in an α-Gal epitope-expressing cell. To achieve this, we used a 0.63-kb IRES sequence that enables simultaneous expression of at least two proteins from a single mRNA [11,12,13]. Subsequently, increased expression of EndoGalC accelerated the digestion of the α-Gal epitope on the cell surface. This reverse correlation between EndoGalC and α-Gal epitope expression was confirmed in the current study (see Figure 2D). Notably, because EndoGalC is produced by bacteria, such as *C. perfringens*, rather than mammalian cells, there is a possibility that EndoGalC expression in mammalian cells would affect cellular properties such as proliferation rate, cell behavior (including cell migration and differentiation), and cellular metabolism. Watanabe *et al*. [16] attempted to address this problem by producing transgenic mice that exhibited systematic expression of EndoGalC. Their results showed that although mice at the newborn stage transiently exhibited growth retardation with abnormal keratinogenesis, those at adult stages gained weight normally and showed normal skin formation. Their internal organs were also normal, and the reproductive ability was not impaired. The same research group later demonstrated that mouse NIH3T3 cells, transfected with an EndoGalC-expression vector, exhibited greater proliferative activity than the untransfected parental cells [17]. Therefore, it is likely that the EndoGalC-expressing cells proliferate faster than intact cells. Therefore, careful attention should be paid to examine whether cellular behavior (including cell proliferation) is altered before and after introducing an EndoGalC-expression vector.

**Figure 5:**
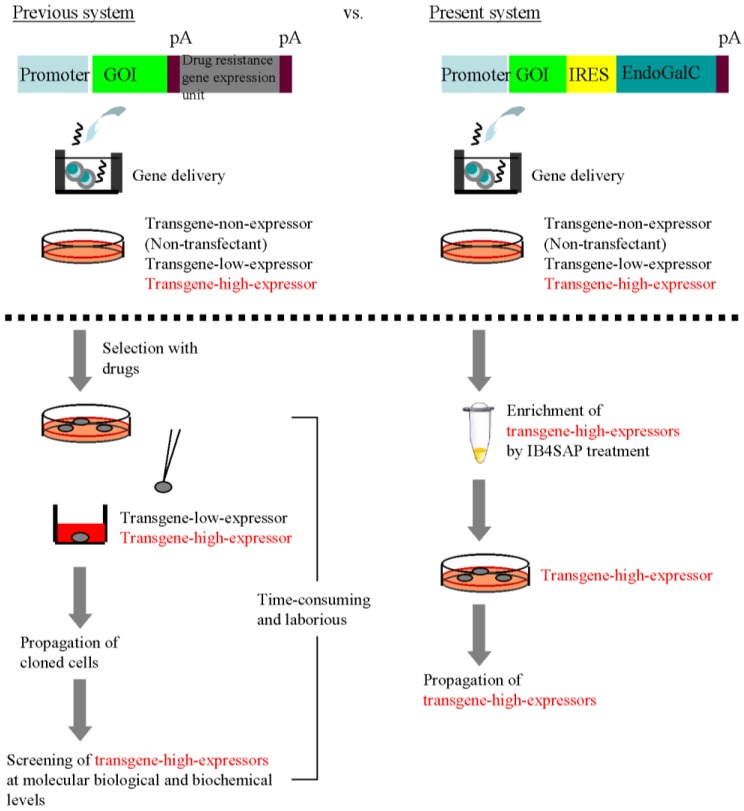
Comparison between the previous system for the cloning of recombinant cells and our present EndoGalC/targeted toxin-based system.

The most excellent property of this system appears to be simple acquisition of transgene high-expressors without drug selection and subsequent molecular biological and biochemical screening of isolated clones. In the traditional cloning approach for isolating transgene high-expressors, the isolation of drug-resistant cells and subsequent characterization of individual clones that have been clonally propagated are essential steps (Figure 5, previous system). Characterizing the isolated clones at the molecular biological and biochemical levels is time-consuming and laborious. In contrast, our EndoGalC/IB4SAP-based system does not require either drug selection or subsequent characterization of the isolated clones (Figure 5, current system). The colonies that survive after transfection of an EndoGalC-expressing vector and subsequent IB4SAP treatment should strongly express the GOI. The results presented in this study prove our proposed hypothesis (Figure 1). Our concern regarding this new system is the procedure to be used to eliminate unwanted cells, which are likely to be untransfected cells escaping from IB4SAP-mediated cell death. However, we experienced a very low incidence of this issue [Table 1; Figure 4B(**e’’**), arrowhead]. Because these cells still express the α-Gal epitope on their surface, they can be eliminated by repeated IB4SAP treatment.

Moreover, this EndoGalC/IB4SAP-based system for the acquisition of transgene high-expressors would be particularly valuable for researchers who wish to perform large-scale production of therapeutically important recombinant proteins (e.g., immunoglobulins) by using mammalian cells. In this case, the drug-free selectable cultivation of cells with high transgene expression is in great demand. To test whether our system can address this demand, we are currently attempting to produce recombinant proteins that can be secreted into the medium by introducing a gene encoding for secreted alkaline phosphatase (SEAP). This system is also applicable to other α-Gal epitope-expressing cells. For example, we successfully obtained transgene high-expressors in mouse NIH3T3 cells using this technology (data not shown). Furthermore, this system appears to be useful in the xenotransplantation field. We have recently succeeded in isolating porcine cells with greatly reduced expression of the α-Gal epitope after transfection with a vector expressing small interfering RNA (siRNA) for α-GalT [18]. We showed that when these isolated siRNA-expressing cells were used as donor cells for somatic cell nuclear transfer (SCNT) experiments in pigs, the resulting cloned blastocysts exhibited a significant reduction in α-Gal epitope expression. This result has encouraged us to plan studies for producing α-Gal epitope-negative cloned piglets by using SCNT.

## 3. Experimental Section

### 3.1. Cell Cultures

The PEFs used throughout this study were primarily cultured from male fetuses of Clawn miniature swine (Japan Farm, Ltd., Kagoshima, Japan) at 30 day after insemination. Cells were grown in PEF culture medium containing Dulbecco’s modified Eagle’s medium/Ham’s F12 (DMEM/Ham’s F12; #124; Wako Pure Chemical Industries, Ltd., Osaka, Japan), 10% fetal bovine serum (FBS), and 1× antibiotic-antimycotic solution (#A5955; Sigma-Aldrich Co. Ltd., St. Louis, MO, USA) at 38.5 °C in a humidified atmosphere of 5% CO_2_ in air. The cells were passaged 3–4 times and then frozen. Frozen cells were thawed and passaged for 7–13 generations prior to transfection.

THEPNBS cells were derived from PEFs transfected with neomycin (*neo*)-, puromycin (*pac*)-, hygromycin B (*hph*)-, blasticydine S (*Sh ble*)-, and zeocin (*zeo*) resistance genes and, therefore, exhibited multidrug resistance against G418, puromycin, hygromycin B, blasticydine S, and zeocin [14]. These cells also express the EGFP and the tdTomato fluorescent protein (red fluorescence). Cells were maintained as PEFs, with the additional use of multiple drugs.

### 3.2. Vector Construction

For construction of an EndoGalC expression plasmid that confers simultaneous expression of a gene of interest and EndoGalC in a transfected cell, an 1.53-kb fragment consisting of a 0.9-kb EGFP cDNA (Clontech Lab.) and a 0.63-kb IRES was inserted upstream of the 3-kb EndoGalC gene [6] in pCAG/EndoGalC [8]. The resulting construct was termed pCEIEnd (Figure 2B), in which expression of both EndoGalC and EGFP are under the control of a strong ubiquitous promoter, CAG (cytomegalovirus enhancer with chicken β-actin promoter) [19]. EndoGalC (GT + Endo) contains a cytoplasmic tail and a transmembrane domain; a stem region of pig α-GalT cDNA was inserted upstream of the full-length EndoGalC gene [6]. Therefore, the EndoGalC protein expressed in cells is retained at the cell membrane where it is expected to exert enzymatic activity. pCEIEnd also contains the backbone of pBluescript SK(-) (Stratagene, La Jolla, CA, USA). pCZIEnd (Figure 4A) was constructed by replacing the EGFP cDNA with the lacZ gene. pCE-29 (Figure 2B; [20]) carrying a CAG promoter-driven EGFP expression unit as well as the pBluescript SK(-) backbone was used as a control.

The fidelity of these plasmids was confirmed by restriction enzyme analysis and sequencing. Plasmids amplified in *Escherichia coli* (DH5α) were purified using the Qiagen Plasmid DNA Isolation Midi Kit (Qiagen GmbH, Hilden, Germany). Circular plasmids were used for the transient expression assay, whereas plasmids linearized by appropriate digestion enzymes were used for acquisition of stable transfectants.

### 3.3. Experiment 1

To explore the optimal concentrations of AF594-IB4, PEFs recovered from dishes by trypsinization were incubated for 1 h at room temperature in a solution containing various amounts (0.08, 0.4, 2, 10 and 50 μg/mL) of AF594-IB4 (#I21413; Invitrogen, Carlsbad, CA, USA) in Dulbecco’s modified phosphate-buffered saline without Ca^2+^ and Mg^2+^ (PBS[-]; pH 7.4), 2% FBS, and 1 mM CaCl_2_ (hereafter referred to as “PBS/FBS/CaCl_2_”). After incubation, the cells were washed twice with PBS/FBS/CaCl_2_ and then inspected for fluorescence under a fluorescence microscope (BX60; Olympus, Tokyo, Japan). Micrographs were taken using a digital camera (FUJIX HC-300/OL; Fuji Film, Tokyo, Japan) attached to the fluorescence microscope and printed using a Mitsubishi digital color printer (CP700DSA; Mitsubishi, Tokyo, Japan). The specificity of IB4 for the α-Gal epitope was confirmed by the abolition of lectin staining in the presence of 50 mM galactose (Sigma-Aldrich Co. Ltd.). Briefly, AF594-IB4 (10 μg/mL) in PBS/FBS/CaCl_2_ was first mixed with 100 mM galactose with a ratio of 1:1 (v/v) for 2 h at room temperature. Cells were then incubated with the mixture for 1 h at room temperature prior to fluorescence observation.

Transient transfection of PEFs with circular plasmids was performed with a nucleofection system (Lonza GmbH, Wuppertal, Germany), as previously described [21]. A schematic flowchart of this experiment is shown in Figure 2B. Briefly, 10 μL of a solution containing circular pCEIEnd or pCE-29 DNA (6 μg) was mixed with 90 μL of the Nucleofector Solution (#11668-027; Lonza GmbH), which was then mixed with PEFs (5 × 10^5^) for transfection. After transfection, cells were cultured in gelatin-coated 60-mm tissue culture dishes (#4020-020; Iwaki Co. Ltd., Tokyo, Japan) in PEF culture medium at 38.5 °C for 2 days. Cells harvested by trypsinization were subjected to cytochemical staining with 5 μg/mL of AF594-IB4, as described above.

### 3.4. Experiment 2

Transfection was performed as described in Experiment 1, except that linearized plasmid DNA was used. The schematic flowchart of this experiment is shown in Figure 3A. Briefly, linearized pCEIEnd DNA (6 μg) was mixed with PEFs (5 × 10^5^) in the Nucleofector Solution (total volume of 100 μL). After transfection, cells were cultured in gelatin-coated 60-mm tissue culture dishes in PEF culture medium at 38.5 °C, and after 4–6 days, the cells were equally divided into 2 sets. One set was treated with IB4SAP (#IT-10; Advanced Targeting Systems Inc., San Diego, CA, USA), whereas the other set was treated with SAP (#PR-01; Advanced Targeting Systems Inc.) as a negative control. For IB4SAP treatment, cells were incubated at 37 °C for 2 h in a solution (20 μL) containing 80 μg/mL of IB4-SAP in PBS/FBS/CaCl_2_. For SAP treatment alone, cells were incubated at 37 °C for 2 h in a solution (20 μL) containing 80 μg/mL of SAP in PBS/FBS/CaCl_2_. The treated cells were directly returned to a 60-mm dish containing normal PEF culture medium and cultured for an additional 1 or 3 weeks. In the IB4SAP-treated group, emerging colonies picked using a small paper disc (3 MM Whatman paper; width × length, 3 × 3 mm) that was dipped in 0.125% trypsin/0.01% EDTA were directly transferred into a gelatin-coated 48-well plate (#3830-048; IWAKI Co. Ltd.) containing PEF culture medium. Cells were cultured for 10–20 days until confluency. Upon passage, a portion of cells was subjected to cytochemical staining with AF594-IB4, as described in Experiment 1.

### 3.5. Experiment 3

Transfection was performed as described in Experiment 2, except that THEPNBS cells were used. The schematic procedure is shown in Figure 4A. Briefly, linearized pCZIEnd DNA (6 μg) was mixed with THEPNBS cells (5 × 10^5^) in the Nucleofector Solution. After transfection, the cells were split in a ratio of 1:10; the former (1/11 of total cells) was seeded in a gelatin-coated 30-mm tissue culture dish, and the latter cells (10/11 of total cells) were seeded in a gelatin-coated 60-mm tissue culture dish. After 2 days, cells in 30-mm tissue culture dishes were fixed with 2% paraformaldehyde in PBS(-) for 5 min at room temperature, and then stained for lacZ activity in the presence of X-Gal (substrate for lacZ) by using the X-Gal Staining Assay Kit (Genlantis Inc, Abingdon, UK). The cells in 60-mm tissue culture dishes were harvested after 4–6 days by trypsinization and then treated with IB4SAP as described in Experiment 2. The treated cells were split 1:10; the former was seeded in a gelatin-coated 30-mm tissue culture dish, and the latter was seeded in a gelatin-coated 60-mm tissue culture dish. Two weeks after the IB4SAP treatment, cells in 30-mm tissue culture dishes were subjected to the cytochemical staining for lacZ activity, as described above. Emerging colonies in 60-mm tissue culture dishes were picked using the paper method described in Experiment 2 and were propagated for cell storage and confirmation of lacZ activity.

### 3.6. Image Analysis

Fluorescence in cells stained with AF594-IB4 was recorded using a digital camera, and the image analysis was performed as previously described [8]. Since cytoplasmic fluorescence for both EGFP and AF594 was noted in cells transfected with either pCE-29 (control) or pCEIEnd (experiment), the intensity of fluorescence (green or red) throughout a cell was measured using a program set with Adobe Photoshop version 5 (Adobe System, Inc., Seattle, WA, USA). pCE-29-transfected cells stained with AF594-IB4 in the presence of 50 mM galactose were used as controls. Results from at least more than 12 cells randomly selected from each group were analyzed and plotted.

## 4. Conclusions

In conclusion, we have shown here that the EndoGalC/IB4SAP-based target toxin system is useful for isolating transgene high-expressors with relative ease. This method would be especially helpful for the large-scale production of recombinant proteins as well as for the acquisition of genetically engineered multidrug-resistant cells.
